# m^6^A RNA methylation regulator heterogeneous nuclear ribonucleoprotein C: A prognostic biomarker for invasive ductal carcinoma validated through Mendelian randomization and transcriptome analyses

**DOI:** 10.1097/MD.0000000000044733

**Published:** 2025-10-10

**Authors:** Yibei Wang, Quhuan Li, Dongshan Sun, Ning Yang, Ying Kong, Yue Shen, Fengxia Zhang

**Affiliations:** a School of Bioscience and Bioengineering, South China University of Technology, Guangzhou, China; b Department of Nephrology, First Affiliated Hospital of Gannan Medical University, Ganzhou, Jiangxi, China.

**Keywords:** *HNRNPC*, invasive ductal carcinoma, m^6^A RNA methylation, Mendelian randomization, transcriptome analysis

## Abstract

Although aberrant N6-methyladenosine (m^6^A) RNA methylation has been linked to oncogenesis and tumor progression, the association between the deregulation of m^6^A regulators and invasive ductal carcinoma (IDC), the predominant subtype of breast cancer, remains unclear. In this study, we sought to determine the function of m^6^A RNA methylation regulators in IDC, with a particular focus on assessing their potential as prognostic biomarkers. To identify dysregulated m^6^A RNA methylation regulators, we systematically analyzed 656 samples from patients with IDC and 81 normal samples from The Cancer Genome Atlas (TCGA) database, and Cox univariate, LASSO-Cox regression, and stepwise regression analyses were conducted to construct a risk-prediction model for determining patient prognosis. Subsequently, we evaluated the prognostic value of the risk signature in IDC and assessed potential biological associations based on clinical survival analyses, examination of publicly available immunohistochemical staining data from the Human Protein Atlas, and two-sample Mendelian randomization. Among the IDC samples, we identified 12 m^6^A RNA methylation regulators characterized by significant dysregulation. Subsequently, a 4-gene signature comprising heterogeneous nuclear ribonucleoprotein C (*HNRNPC*), YTH domain-containing family proteins 2 and 3 (*YTHDF2/3*), and RNA-binding motif protein 15B (*RBM15B*) was constructed using machine learning algorithms. This signature was established to be an independent prognostic factor, particularly in patients with early stage IDC, and within the signature, *HNRNPC* was identified as a pivotal gene, the expression levels of which were demonstrated to be causally associated with the risk of IDC. On the basis of our findings in this study, we established a prognostic signature for IDC and identified a causal association between the expression of the signature gene *HNRNPC* and IDC risk. These findings indicate that m^6^A RNA methylation regulators could serve as molecular biomarkers for IDC and contribute to guiding therapeutic strategies.

## 1. Introduction

Among the most common malignancies affecting women globally, breast cancer ranks within the top 10.^[1]^ According to the 2022 data released by the International Agency for Research on Cancer (IARC) under the World Health Organization, over 2.3 million new breast cancer cases were reported worldwide, accounting for 23.8% of all cancer cases in women.^[2]^ On the basis of histology and molecular features, breast cancer can be classified into a number of different subtypes,^[3]^ among which, invasive ductal carcinoma (IDC), the most prevalent invasive breast cancer that invades the ducts and is present in the stroma, accounts for 55% of breast cancer incidence upon diagnosis.^[4,5]^ Consequently, from the perspective of precision medicine, there is an imperative to identify specific biomarkers that can contribute to enhancing diagnosis and treatment and aid in the prediction of prognosis.

In many eukaryotic species, N6-methyladenosine (m^6^A), in which the N6 position of adenosine is methylated, is the most prevalent internal modification of messenger RNAs (mRNAs) and long noncoding RNAs.^[6,7]^ The modulation of m^6^A RNA methylation dynamics is governed by the interplay among methyltransferases (referred to as “writers”), binding proteins (“readers”), and demethylases (“erasers”). Writers, which include methyltransferase-like 3 (METTL3),^[8]^ METTL14,^[9]^ METTL16,^[10]^ zinc finger CCCH domain-containing protein 13 (ZC3H13),^[11]^ RNA-binding motif protein 15 (RBM15), RBM15B,^[12,13]^ Vir-like m^6^A methyltransferase associated (VIRMA/KIAA1429),^[14]^ and Wilms Tumor 1-Associating Protein (WTAP),^[15]^ convey methyl groups from the *S*-adenosylmethionine donor to the adenine base.^[16]^ Subsequently, this m^6^A methylation is recognized by readers, such as heterogeneous nuclear ribonucleoprotein C (HNRNPC), heterogeneous nuclear ribonucleoprotein A2-B1 (HNRNPA2B1),^[17,18]^ YTH domain family proteins 1 (YTHDF1), YTHDF2, YTHDF3,^[19,20]^ YTH domain-containing proteins 1 (YTHDC1),^[21]^ and YTHDC2.^[22]^ This dynamic m^6^A methylation can, however, be reversed by the erasers, among which are fat mass and obesity-associated protein (FTO)^[23]^ and α-ketoglutarate dependent dioxygenase 5 (ALKBH5).^[24]^ Although further m6A RNA methylation regulators, such as RBMX,^[25]^ have recently been identified, owing to a lack of corroborating literature, the aforementioned 17 proteins are considered the most canonical m^6^A RNA methylation regulators.

The findings of recent research have indicated that m^6^A RNA modification plays a pervasive role in a number of mRNA-associated processes, including the maintenance of stability, facilitating translation, regulating transport, influencing splicing, and determining localization. Moreover, such modification has been established to influence a range of physiological functions, including the self-renewal and differentiation of embryonic stem cells, regulation of circadian rhythms, and spermatogenesis.^[6,7]^ An accumulating body of evidence indicates that the abnormal expression of m^6^A is associated with multiple human diseases, including a range of cancers.^[16,26]^ In this regard, certain risk signatures characteristic of abnormal m^6^A RNA methylation regulators have been established to be correlated with overall survival rates, thereby indicting their potential application as valuable prognostic biomarkers for predicting the outcomes in different cancer types, including cervical cancer,^[27]^ head and neck squamous cell carcinoma,^[28]^ gliomas,^[29]^ lung adenocarcinoma,^[30]^ and colorectal cancer.^[31]^ With respect to IDC, the findings of recent studies have revealed the aberrant m^6^A RNA methylation in CD4^+^ T helper cells, and have provided evidence that manipulating this methylation can potentially impede disease transmission under different conditions.^[32]^ However, further research is necessary to establish the prognostic value of risk signatures based on aberrant m6A RNA methylation regulators in IDC.

**Figure 1.**
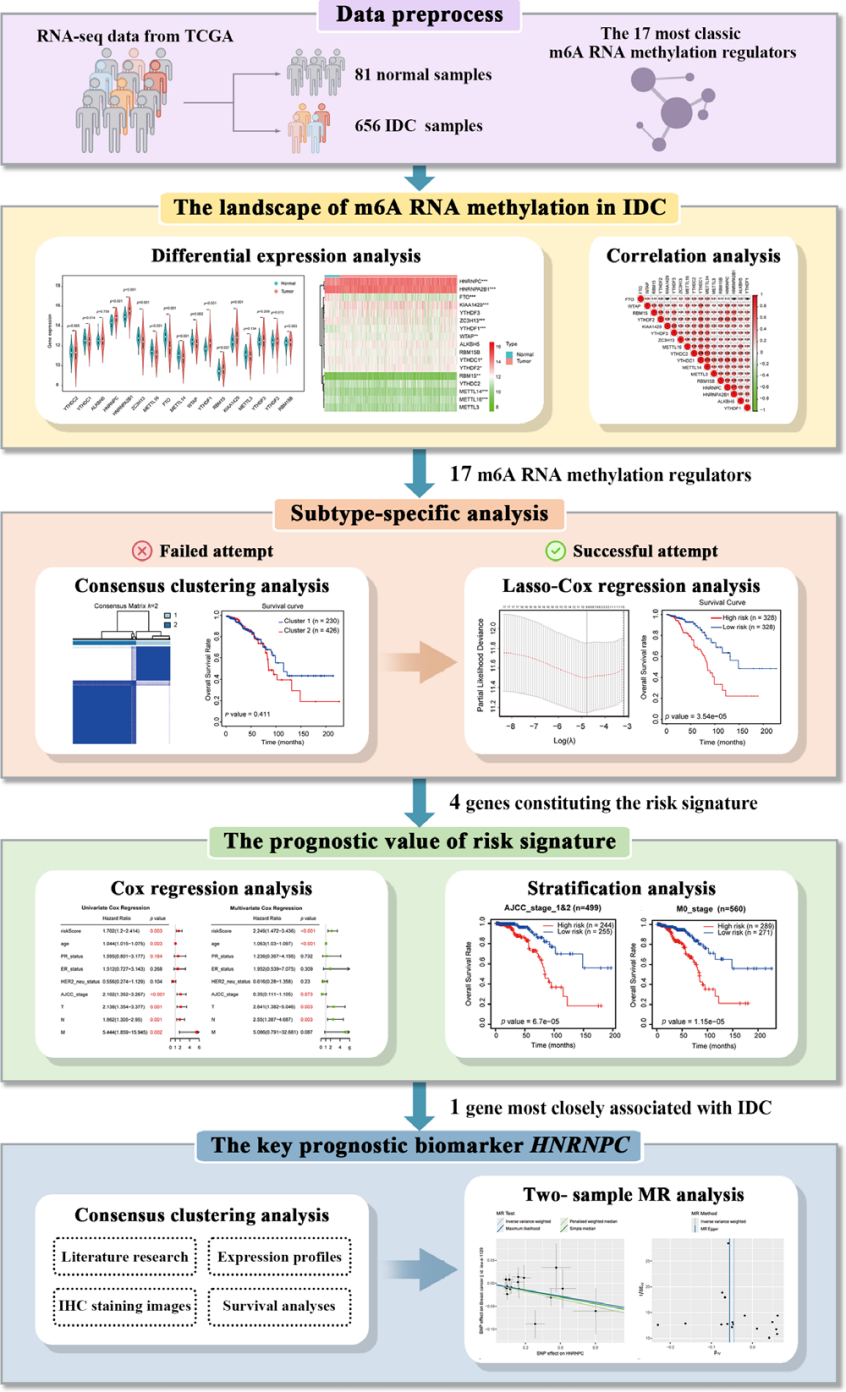
Schematic diagram of the research design and analysis.

Mendelian randomization (MR), a valuable tool that is widely adopted in epidemiological research, can be used to accurately evaluate the likelihood of causal relationships between exposures and outcomes.^[33]^ To circumvent the issues of reverse causality and potential confounding influences, MR analysis employs genetic variants as instrumental variables (IVs), and consequently, this technique is uninfluenced by environmental risk factors and is typically applied prior to the advancement of disease.^[34]^ In previous studies on breast cancer, MR has been successfully applied to identify putative causal genes and actionable druggable targets. For example, farnesyl diphosphate synthase has been demonstrated to be positively correlated with the risk of breast cancer,^[35]^ and raloxifene, which targets microtubule-associated protein tau, has been linked to a 35% reduction in breast cancer risk.^[36]^ In the present study, using aggregate statistics derived from previous genome-wide association studies (GWAS) and transcriptomic information, we conducted MR analysis to determine the role of m^6^A RNA methylation in the initiation and progression of IDC and to enhance the prediction of prognosis based on the expression of m^6^A RNA methylation regulators (Fig. 1).

Given the prevalence and pronounced impact of IDC, we meticulously examined the expression of the aforementioned 17 most canonical m^6^A RNA methylation regulators in 656 patients with IDC using data derived from The Cancer Genome Atlas (TCGA) database, on the basis of which, we constructed a risk-prediction model for predicting risk signature, using a machine learning approach, entailing Cox univariate, least absolute shrinkage and selection operator (LASSO)-Cox regression, and stepwise regression analyses. Subsequently, we undertook an MR study to assess the prognostic value of the risk signature in IDC and investigated the causal association between *HNRNPC*, a key gene among the prognostic biomarkers, and breast cancer.

## 2. Materials and methods

### 2.1. Data sources and preprocessing

RNA sequencing (RNA-seq) transcriptome data and the corresponding clinical datasets were extracted from the TCGA database (https://cancergenome.nih.gov/), from which count data for the RNA transcriptome were downloaded. Having excluded those cases with incomplete survival data and those with patient survival periods of <1 month postdiagnosis, we obtained 696 IDC samples and 81 normal samples for analysis based on the following 3 considerations: the predominance of non-tumor-related causes of mortality, incomplete baseline molecular profiling in rapidly progressive cases, and violation of the proportional hazards assumption in Cox regression modeling. The TCGA RNA-seq data had initially undergone official standardized preprocessing to minimize batch effects from different sequencing centers, and to avoid <correction, we refrained from applying additional batch-effect adjustments. For sequencing analysis, we also downloaded information on clinicopathological features, including age, American Joint Committee on Cancer (AJCC) cancer stage, tumor node metastasis (TNM) stage, and the status of estrogen receptor (ER), progesterone receptor (PR), and human epidermal growth factor receptor 2 (HER2). In addition, to conduct external validation of the risk signature, we employed the following 3 independent datasets obtained from the Gene Expression Omnibus (GEO) database (https://www.ncbi.nlm.nih.gov/geo/): GSE61304, sourced from Singapore, comprising 58 IDC samples with available survival information; GSE42568, originating from Ireland, comprising 104 breast cancer samples with survival data; and GSE7390, derived from Canada, comprising 198 breast cancer samples with survival information.

### 2.2. Selection of m^6^A RNA methylation regulators

With reference to previous research on m^6^A RNA methylation, we selected 17 genes for methylation regulator analysis, namely, *METTL3, METTL14, METTL16, RBM15, RBM15B, YTHDC2, YTHDC1, YTHDF1, YTHDF2, YTHDF3, HNRNPA2B1, HNRNPC, WTAP, KIAA1429, ZC3H13, FTO*, and *ALKBH*,^[8–24]^ for which the expression profiles and corresponding clinical information were obtained. A logarithmic function conversion was performed using an expression matrix.

### 2.3. Differential expression and correlation analyses

To assess disparities in the expression of m^6^A RNA methylation regulators between normal and cancer samples, we used the Wilcoxon test, and generated violin plots and heatmaps to visualize the differential expression of m^6^A RNA methylation regulators using the R packages vioplot and pheatmap, respectively. Thereafter, Pearson correlation analysis was performed to determine the relationships between distinct m^6^A RNA methylation regulators, with the results being visualized in correlation heatmaps generated using the R package corrplot.

### 2.4. Consensus clustering analysis

Having initially excluded data for the normal samples, consensus clustering analysis was performed based on the expression of m^6^A RNA methylation regulators using the R package ConsensusClusterPlus. In addition, to determine variations in overall survival among distinct patient clusters, we used the Kaplan–Meier method and log-rank test, and the distribution of the clinicopathological features of different clusters was visualized using a heatmap.

### 2.5. Generation and predictive performance of the risk signature

To determine the prognostic value of the 17 m^6^A RNA methylation regulators in IDC, we performed univariate Cox regression analysis. Thereafter, LASSO-Cox regression analysis was conducted to facilitate the construction of a prediction model for the risk signature of overall survival, and stepwise regression was applied to obtain the optimal model. On the basis of median risk scores calculated using the model, IDC samples were classified into low- and high-risk groups for which survival disparities were evaluated using the Kaplan–Meier method and log-rank test, with receiver operating characteristic (ROC) curves being used to assess the accuracy of the model in predicting overall survival at 1, 3, and 5 years. To evaluate the predictive performance of the established risk signature, for comparative purposes, we used 4 IDC models, 10 breast cancer models, and 5 triple-negative breast cancer models using TCGA data.^[37–55]^ These models cover a range of biological processes and features, including glycolysis, coagulation, lipid metabolism, ferroptosis, immunity, and the tumor microenvironment.

### 2.6. Analysis of risk signature independence

To establish whether the risk signature could serve as an independent prognostic factor, we performed univariate and multivariate Cox regression analyses in conjunction with an analysis of other clinicopathological features. The influence of the distribution of clinicopathological features in the low- and high-risk groups of patients with IDC was estimated using the chi-square test. To further determine the prognostic value of the risk signature, differences in the overall survival of patients in these 2 groups were analyzed using the Kaplan–Meier method and log-rank test having stratified IDC samples with respect to age; AJCC, T, N, and M stage; and ER, PR, and HER2 status.

### 2.7. Selection of key prognostic biomarkers

On the basis of the modeling outcomes, we used the Kaplan–Meier method and log-rank tests to analyze survival disparities with respect to expression of the candidate genes, and examined the pathological images of these genes obtained from the Human Protein Atlas (HPA) database (https://www.proteinatlas.org/; accessed on August 28, 2024). Given that these images are observational and illustrative in nature and were not based on experiments conducted in the present study, their use constitutes secondary validation rather than primary experimental confirmation. By performing this analysis, we were able identify the key and representative genes among the m^6^A RNA methylation regulators.

### 2.8. Mendelian randomization

To investigate any causal links between hub genes among the m^6^A RNA methylation regulators and IDC, we used the R package TwoSampleMR for a two-sample MR approach. All data used in the MR analysis were extracted from the accessible OpenGWAS database (https://gwas.mrcieu.ac.uk), with the samples in question being exclusively those of individuals of European descent. A comprehensive exposure dataset for HNRNPC can be found in the GWAS catalog identified by the dataset ID “prot-a-1364.” Similarly, a dataset pertaining to the outcome variable (i.e., breast cancer) is registered with the ID “ieu-a-1129” and “ebi-a-GCST004988.” Given the limited number of single-nucleotide polymorphisms (SNPs) linked to any particular gene at the conventional GWAS significance level (5e−08), we used a more lenient *P*-value threshold of 5e−06. This adjustment was performed to ensure a sufficient number of SNPs to serve as IVs. To mitigate the impact of linkage disequilibrium and maintain the independence of variants, the linkage disequilibrium window was defined as 10,000 kb with an *r^2^* threshold of 0.001. To precisely assess causal relationships and account for the influence of horizontal pleiotropy, we employed a range of complementary MR detection techniques, including inverse variance weighting (“IVW”), “MR Egger,” “Simple median,” “Penalized weighted median,” and “Maximum likelihood.” Following the assessments of heterogeneity and pleiotropy, scatter, forest, and funnel plots were generated to visually depict the outcomes of the MR analysis and we evaluated the robustness of our findings using the leave-one-out cross-validation approach.

### 2.9. Statistical analysis

All data manipulation and analytical procedures were performed using the R software (version 4.2.1). Disparities in gene expression between normal and cancer samples were analyzed using the Mann–Whitney *U* test (Wilcoxon rank-sum test), and the Chi-square test was used to compare and analyze the statistical significance between 2 groups of categorical variables. For survival analysis, we used the R package Survival, whereas Kaplan–Meier survival curves were plotted to illustrate survival differences, and the log-rank test was applied to evaluate the significance of differences in survival times between the 2 groups. All statistical tests were two-sided, and a *P*-value < 0.05 was considered statistically significant.

## 3. Results

### 3.1. The m^6^A RNA methylation landscape in IDC

**Figure 2.**
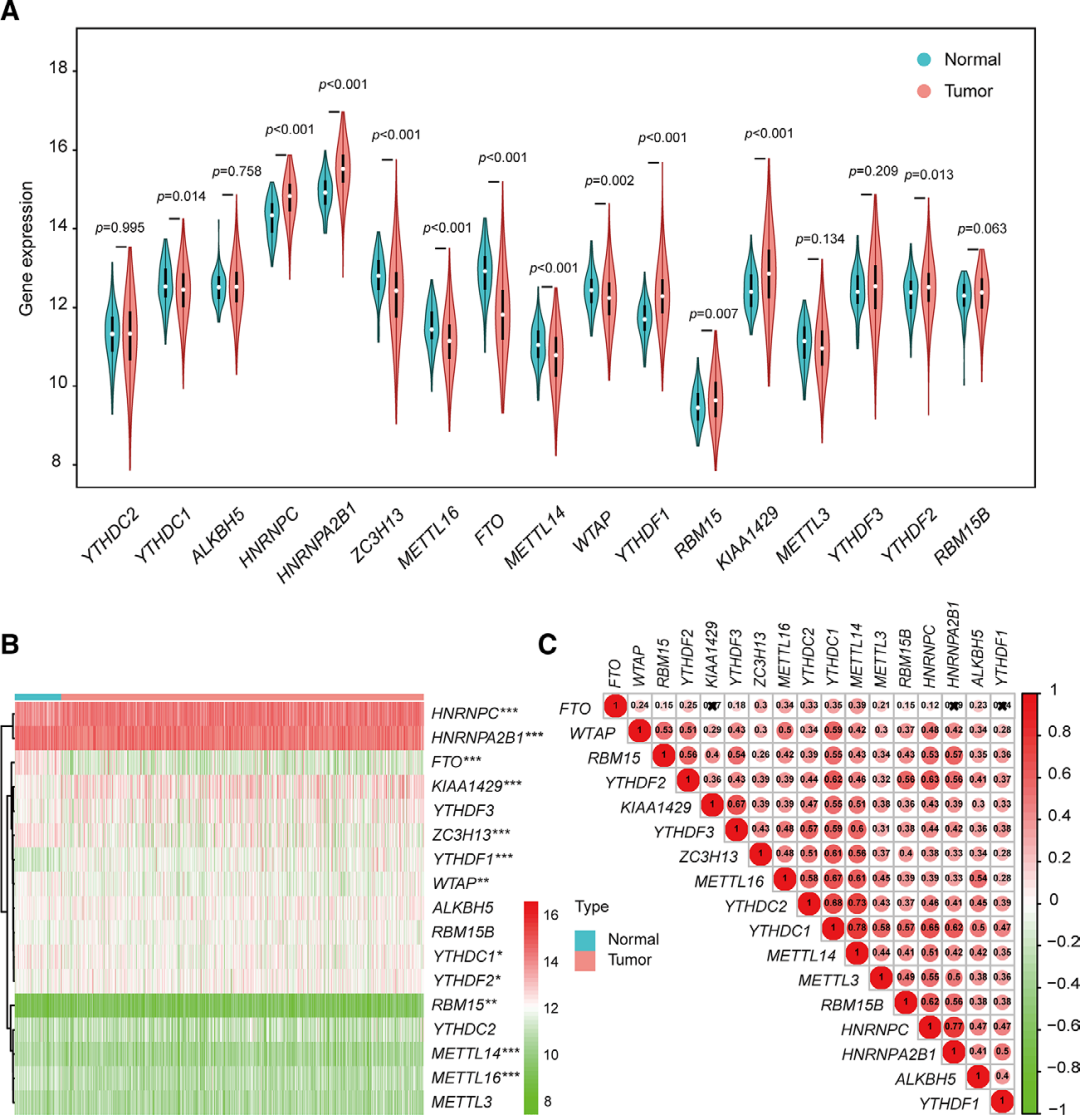
The m^6^A RNA methylation landscape in invasive ductal carcinoma. (A, B) A violin plot (A) and heatmap (B) illustrating the expression of m^6^A RNA methylation regulators in normal and tumor samples. (C) A correlation heatmap illustrating correlations among the m^6^A RNA methylation regulators. *P < .05, **P < .01, ***P < .001.

Given the pivotal role played by m^6^A RNA methylation regulators in oncogenesis and development, we conducted a comparative analysis of the expression of 17 m^6^A RNA methylation regulators in 656 IDC and 81 normal samples obtained from the TCGA database. Compared with normal cases, we detected differences in the expression patterns of m^6^A RNA methylation regulators in patients with IDC (Fig. 2A). In particular, compared with that in the adjacent normal tissues, we observed marked elevations in the expression of *HNRNPC (P* < .001), *HNRNPA2B1 (P* < .001), *YTHDF1 (P* < .001), *RBM15 (P* = .007), *KIAA1429 (P* < .001), and *YTHDF2 (P* = .013) in tumor tissues, whereas normal tissue were found to be characterized by a higher expression of *YTHDC1 (P *= .014), *ZC3H13 (P *< .001), *METTL16 (P *< .001), *FTO (P *< .001), *METTL14 (P* < .001), and *WTAP (P* = .002) (Fig. 2B). Furthermore, our analysis of associations among the m^6^A RNA methylation regulators, based on expression levels, revealed that with the exception of *FTO*, there were strong positive correlations between most regulators, among which *YTHDC1* and *METTL14* showed the highest correlation (*R *= 0.78) (Fig. 2C).

### 3.2. Consensus clustering analysis in patients with IDC driven by the m^6^A RNA methylation regulators

**Figure 3.**
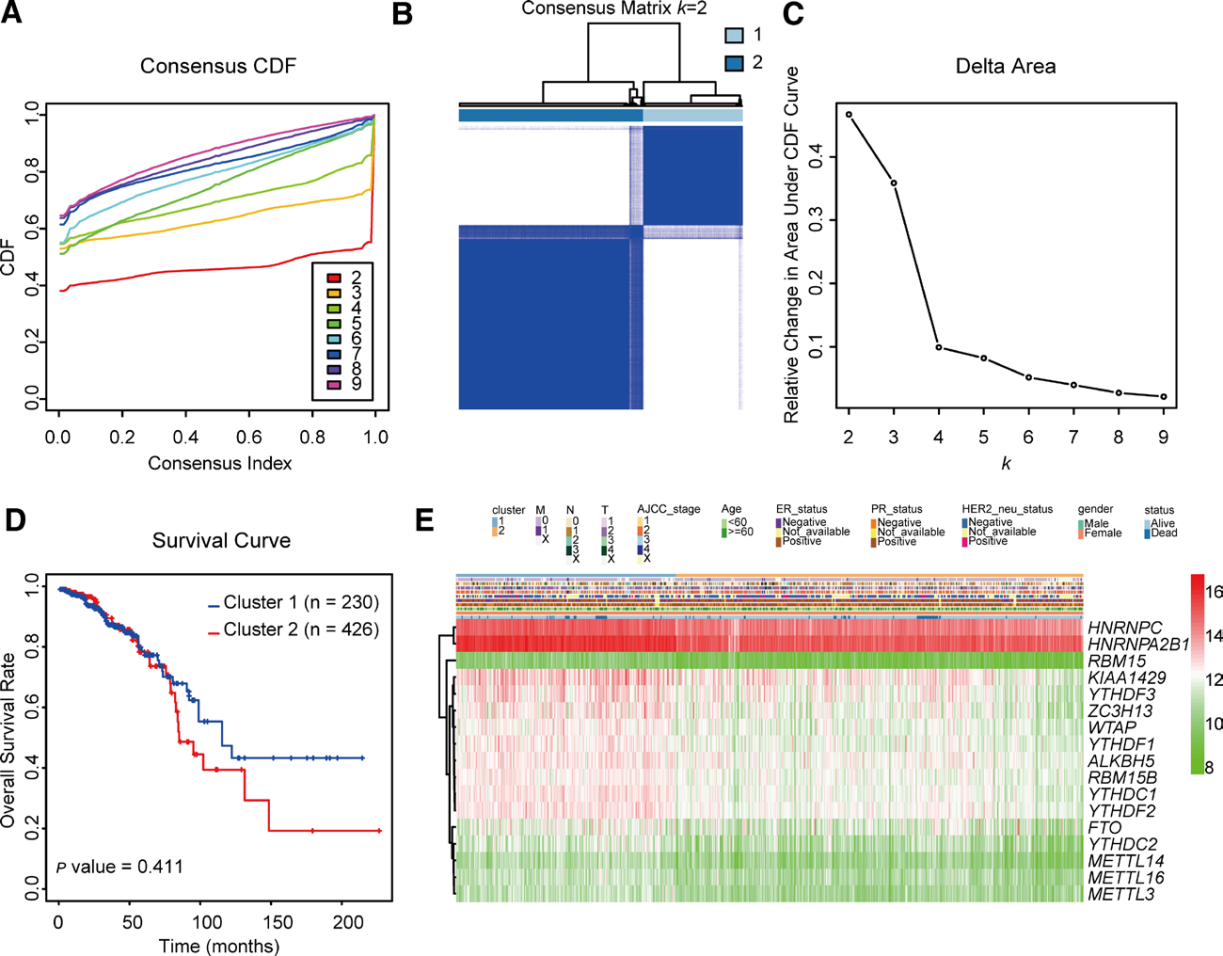
Consensus clustering analysis and different overall survival and clinical features of the clusters. (A) Consensus clustering cumulative distribution function (CDF) for *k* = 2–9. (B) A consensus clustering matrix for *k* = 2. (C) Relative change in the area under the CDF curve for *k* = 2–9. (D) Kaplan–Meier overall survival curves for the 2 clusters. (E) A heatmap showing the expression of the m^6^A RNA methylation regulators and the clinicopathologic features of the tumor samples.

With a view toward differentiating patient subgroups within the IDC cohort based on the expression patterns of the 17 m^6^A RNA methylation regulators, we performed consensus clustering analysis. Given the similarity in expression among the m^6^A RNA methylation regulators, a *k* value of 2 was deemed appropriate for classifying IDC patients into 2 distinct clusters. (Fig. 3A–C). Subsequently, to elucidate the clustering results and assess the associations with clinicopathological features, we conducted overall survival analysis. However, we detected no statistically significant differences between the 2 clusters with respect to survival outcomes (Fig. 3D). Despite the observed upregulation of *HNRNPC, HNRNPA2B1, KIAA1249, YTHDF3, ZC3H13, WTAP, YTHDF1, ALKBH5, RBM15B, YTHDC1, YTHDF2*, and *FTO*, and the downregulation of *RBM15, YTHDC2, METTL14, METTL16*, and *METTL3* in Cluster 1 relative to those in Cluster 2, we detected no substantial variation between the 2 clusters regarding the distribution of clinicopathological parameters (Fig. 3E). These findings accordingly imply that the consensus clustering analysis algorithm may not be appropriate for distinguishing patient subgroups within the IDC cohort. Consequently, it will be necessary to identify alternative bioinformatic methods to determine the subgroups of patients with IDC based on the expression levels of m^6^A RNA methylation regulators.

### 3.3. Prognostic value of a risk signature comprising 4 m^6^A RNA methylation regulators

**Figure 4.**
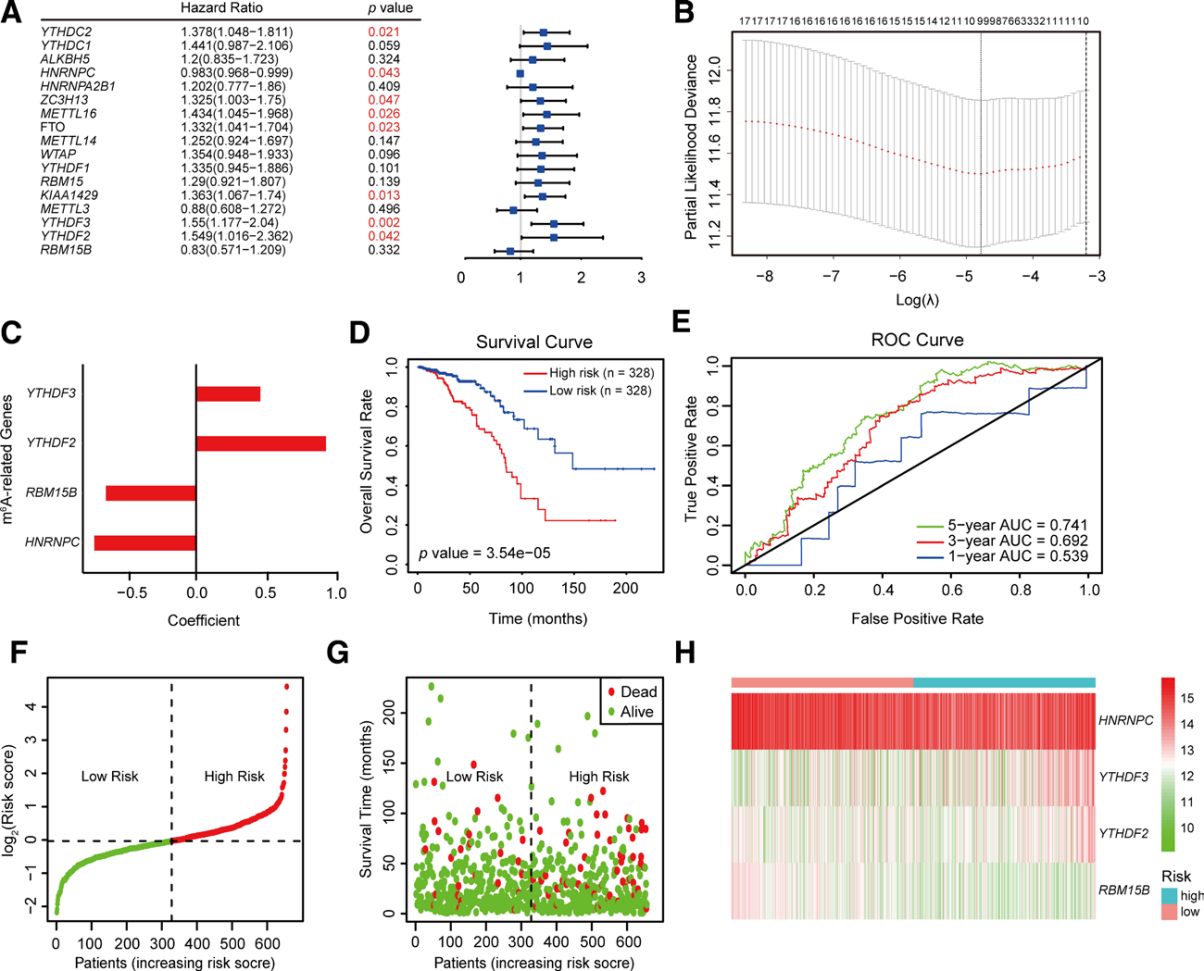
Prognostic value of the risk signature comprising 4 m^6^A RNA methylation regulators. (A) Univariate Cox regression analysis for associations between the 17 m^6^A RNA methylation regulators and overall survival. (B) LASSO regression used to enhance the fit of the model, and cross-validation curves used to select the tuning parameters in the LASSO model. (C) Coefficients of the multivariate Cox regression model constructed using the 4 m^6^A RNA methylation regulators. (D) Kaplan–Meier overall survival curves for the high- and low-risk patients with invasive ductal carcinoma. (E) An ROC curve showing the efficiency of the risk score predictions for 1, 3, and 5 years. The larger the area under the curve (AUC) value, the more accurate is the performance. (F–H) A dot plot and heatmap for the risk scores (F), survival time (G), and expression of 4 m^6^A RNA methylation regulators (H) when the risk score is arranged in increasing order. ROC = receiver operating characteristic.


Given the inconclusive findings regarding consensus clustering, we performed Cox regression analysis with the objective of developing a risk-scoring model that could serve as a novel classification criterion, utilizing the risk score as a more informative indicator for patient stratification. To elucidate the prognostic significance of the 17 m^6^A RNA methylation regulators in IDC, we conducted univariate Cox regression analysis using the data for IDC patients obtained from the TCGA database. Among these, *YTHDC2, ZC3H13, METTL16, FTO, KIAA1429, YTHDF3*, and *YTHDF2* were identified as risk factors for IDC (Fig. 4A). To predict the overall survival of patients with IDC based on the expression of m^6^A RNA methylation regulators, we employed both the LASSO-Cox regression algorithm and stepwise regression method to analyze the 17 candidate genes from the TCGA dataset (Fig. 4B). A risk-associated signature was formulated by selecting 4 of the assessed genes, for which coefficients were determined using multivariate Cox regression analysis (Fig. 4C). The risk score for patients with IDC was determined using the following formula that incorporates the expression levels of the 4 genes:

$$\begin{array}{lll} Risk\;Score= & -0.742\times HNRNPC+0.469\times YTHDF3\; & -0.947\times YTHDF2-0.656\times RBM15B \end{array}$$

To further characterize the associations between the risk signature and the overall survival of patients with IDC, we initially performed survival analysis on groups stratified as high- and low-risk based on the median risk score. Our finding of a significant disparity in the overall survival of patients in the 2 groups (*P* = 3.54e^−05^) was taken to be indicative of a correlation between the risk signature and overall survival (Fig. 4D). On the basis of the construction of ROC curves, we obtained area under the curve (AUC) values of 0.539, 0.692, and 0.741 for the predictive accuracy of the risk scores for 1-, 3-, and 5-year predicted survival, thereby indicating progressively stronger discrimination at longer follow-ups (Fig. 4E). Notably, this performance exceeded that of previously developed prognostic models, including the best-performing IDC-specific models (1-/3-/5-year AUCs: 0.72, 0.642, and 0.592), breast cancer models (1-/3-/5-year AUCs: 0.728, 0.576, and 0.589), and triple-negative breast cancer models, which showed a generally poor predictive capacity for IDC (Fig. S1, Supplemental Digital Content, https://links.lww.com/MD/Q187). Moreover, those patients classified as low risk in this study were found to be characterized by a longer survival than those deemed to be at a high risk. In addition, comparison of the high- and low-risk patients revealed distinct differences in the expression profiles of the 4 signature genes, among which, *YTHDF3* and *YTHDF2* tended to be characterized by an elevated expression in the high-risk group, whereas the expression of *RBM15B* was generally higher in the low-risk group (Fig. 4F–H).

### 3.4. Independence of the m^6^A RNA methylation regulator-based risk signature

**Figure 5.**
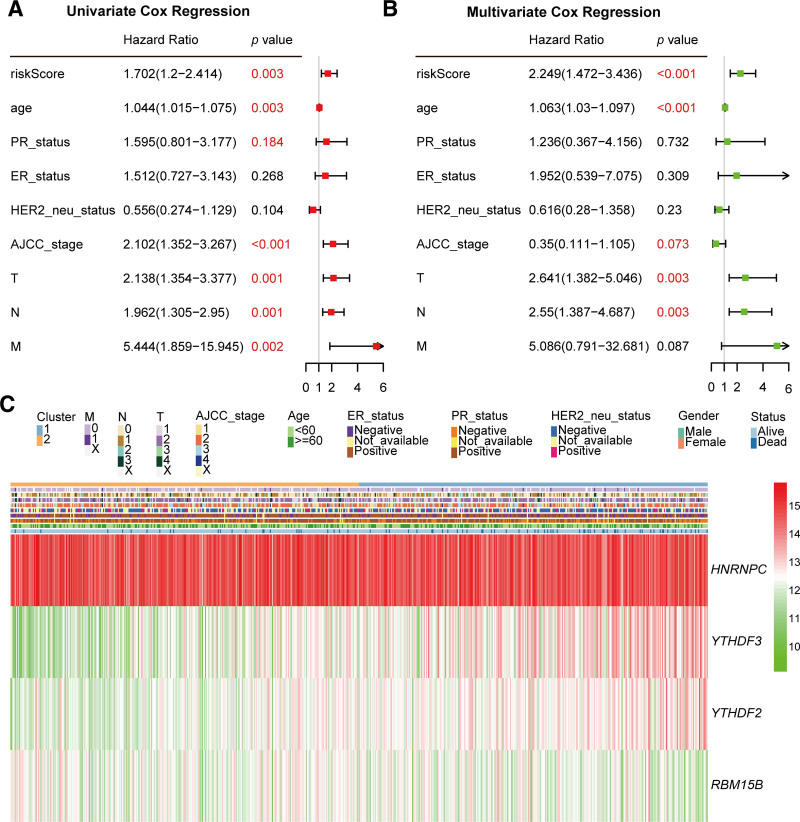
Independence of the risk signature comprising m^6^A RNA methylation regulators. (A, B) Univariate and multivariate Cox regression analysis for the associations between overall survival and clinicopathologic features (including the risk score). (C) A heatmap showing the disparate expression patterns of 4 m^6^A RNA methylation regulators, along with variations in the risk scores and clinicopathological features.

To ascertain whether the risk signature of patients with IDC would serve as an independent prognostic factor relative to other clinicopathological parameters, we conducted Cox regression analysis, for the purposes of which, risk scores and other clinicopathological features were used as covariates. Having excluded patients with incomplete clinicopathological data, the remaining 379 IDC cases were analyzed. Univariate analysis revealed that risk score (*P* = .003), age (*P* = .003), AJCC stage (*P *< .001), T stage (*P* = .001), N stage (*P* = .001), and M stage (*P* = .002) were significantly correlated with the overall survival of IDC patients (Fig. 5A), and subsequent analysis confirmed that risk score (*P* < .001), age (*P* < .001), T stage (*P* = .003), and N stage (*P* = .003) were significantly associated with the overall survival of these patients (Fig. 5B). Furthermore, we investigated whether the distribution of clinicopathological parameters was correlated with the risk signature, the findings of which revealed that among these parameters, only vital status showed a significant association with the risk signature (Fig. 5C). The data presented herein thus provide compelling evidence indicating that the risk signature derived from m^6^A RNA methylation regulators represents an independent prognostic factor.

### 3.5. Stratification analysis for determining the prognostic value of the risk signature

**Figure 6.**
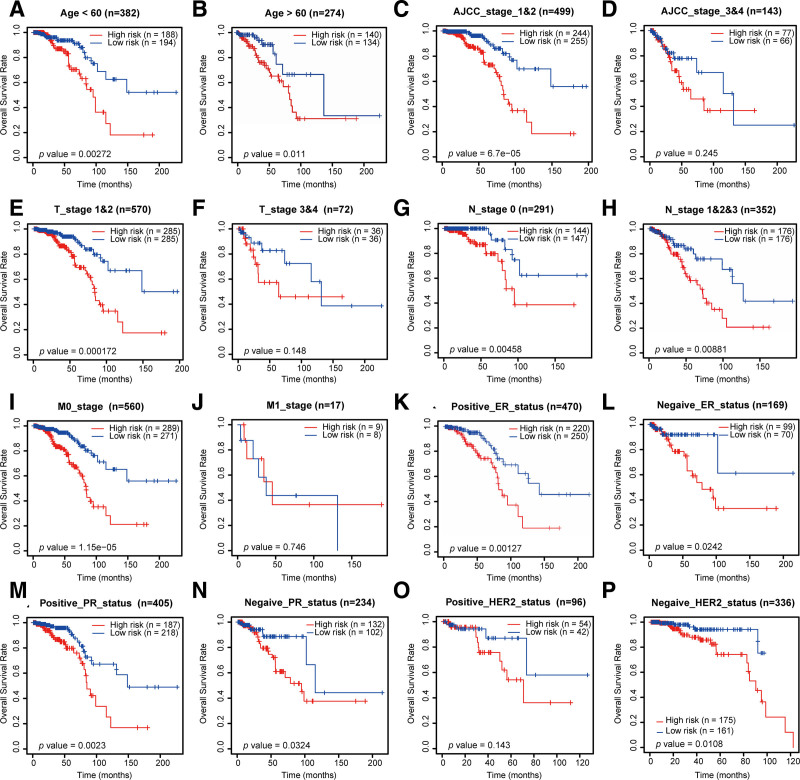
Stratification analysis for the prognostic value of the risk signature. Kaplan–Meier survival curves for the patients with IDC, with age less than 60 yr (A), age more than 60 yr (B), early AJCC stage (C), late AJCC stage (D), early T stage (E), late T stage (F), early N stage (G), late N stage (H), early M stage (I), late M stage (J), positive ER status (K), negative ER status (L), positive PR status (M), negative PR status (N), positive HER2 status (O), and negative HER2 status (P). AJCC = American Joint Committee on Cancer, ER = estrogen receptor, HER2 = human epidermal growth factor receptor 2, IDC = invasive ductal carcinoma, PR = progesterone receptor.

To enhance the applicability of the risk signature, we stratified IDC patients in the TCGA dataset based on different clinicopathological characteristics, and subsequently performed survival analysis on the 2 distinct risk groups thus obtained. With respect to age, N stage, ER status, and PR status, patients in the high-risk group were established to have a shorter overall survival than those in the low-risk group. In addition, compared with patients in the low-risk group, we observed significantly shorter overall survival among high-risk patients with AJCC stage 1 and 2 (*P* = 6.7e^−05^), stage T1 and T2 (*P* = .000114), stage M0 (*P* = 1.15e^−05^), and a negative HER2 status (*P* = .0108), whereas there was no significant disparity between the 2 risk groups with respect to AJCC stages 3 and 4, stages T3 and T4, stage M1, or a positive HER2 status (Fig. 6A–P). In subsequent assessments of the 3 external validation datasets obtained from GEO, Kaplan–Meier survival curve analysis revealed significant differences in overall survival between the high-risk and low-risk groups (*p* values of 0.0021, 0.0025, and 0.006, respectively), thus providing evidence to indicate that the risk signature developed in this study can be applied to effectively differentiate patients based on risk level and shows robust prognostic discrimination (Fig. S2A–C, Supplemental Digital Content, https://links.lww.com/MD/Q187). Furthermore, ROC curves confirmed the predictive accuracy of the risk signature at different time points postdiagnosis (1, 3, and 5 years), thereby highlighting its clinical applicability and robustness among heterogeneous populations (Fig. 2D–F, Supplemental Digital Content, https://links.lww.com/MD/Q187).

### 3.6. Identification of *HNRNPC* as a key prognostic biomarker

**Figure 7.**
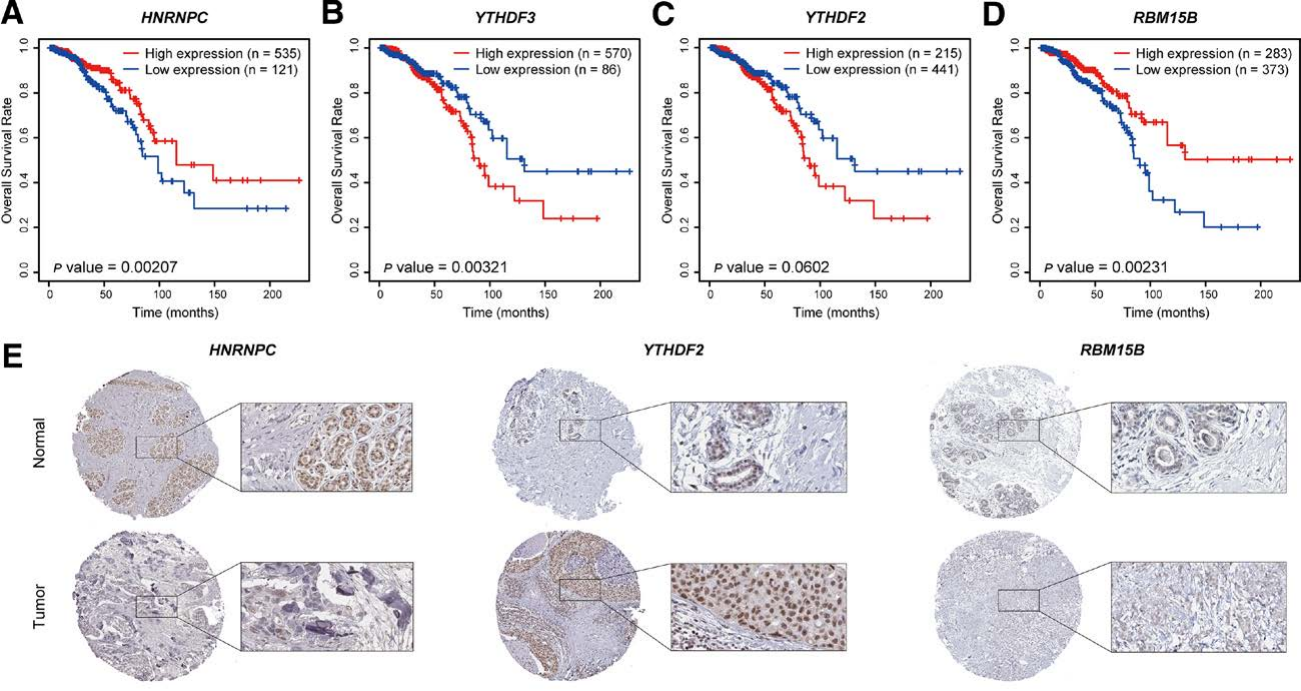
Selection of key prognostic biomarkers. (A–D) Kaplan–Meier curves for high and low expression groups of HNRNPC (A), YTHDF3 (B), YTHDF2 (C), and RBM15B (D) in the TCGA dataset for patients with invasive ductal carcinoma. (E) Immunohistochemical staining images of HNRNPC, YTHDF2, and RBM15B in normal and breast cancer tissues derived from the Human Protein Atlas database. HNRNPC = heterogeneous nuclear ribonucleoprotein C, RBM15B = RNA-binding motif protein 15B, YTHDF2 = YTH domain family protein 2, YTHDF3 = YTH domain family protein 3.

To determine whether the 4 genes constituting the risk signature for IDC function as key genes, we conducted survival analysis for the TCGA cohort, which revealed that a high expression of *HNRNPC (P* = .00207) and *RBM15B (P* = .00231) was associated with a significantly enhanced overall survival, whereas a high expression of *YTHDF3 (P* = .00321) was established to show a significant correlation with reduced survival (Fig. 7A–D). These findings thus indicate that these genes may have notable prognostic relevance for those with IDC, thereby warranting their consideration as potential therapeutic targets or prognostic indicators. Using the HPA database, we assessed the protein expression of the 4 candidate genes among normal and tumor tissues. Although there were no relevant data for *YTHDF3*, assessment of the other 3 proteins revealed that compared with that in breast cancer tissues, the expression of HNRNPC was significantly elevated in normal tissues (Fig. 7E). On the basis of these findings, and similar observations reported in the literature, we identified *HNRNPC* as a pivotal gene in our model.

### 3.7. *HNRNPC* is causally associated with breast cancer

**Figure 8.**
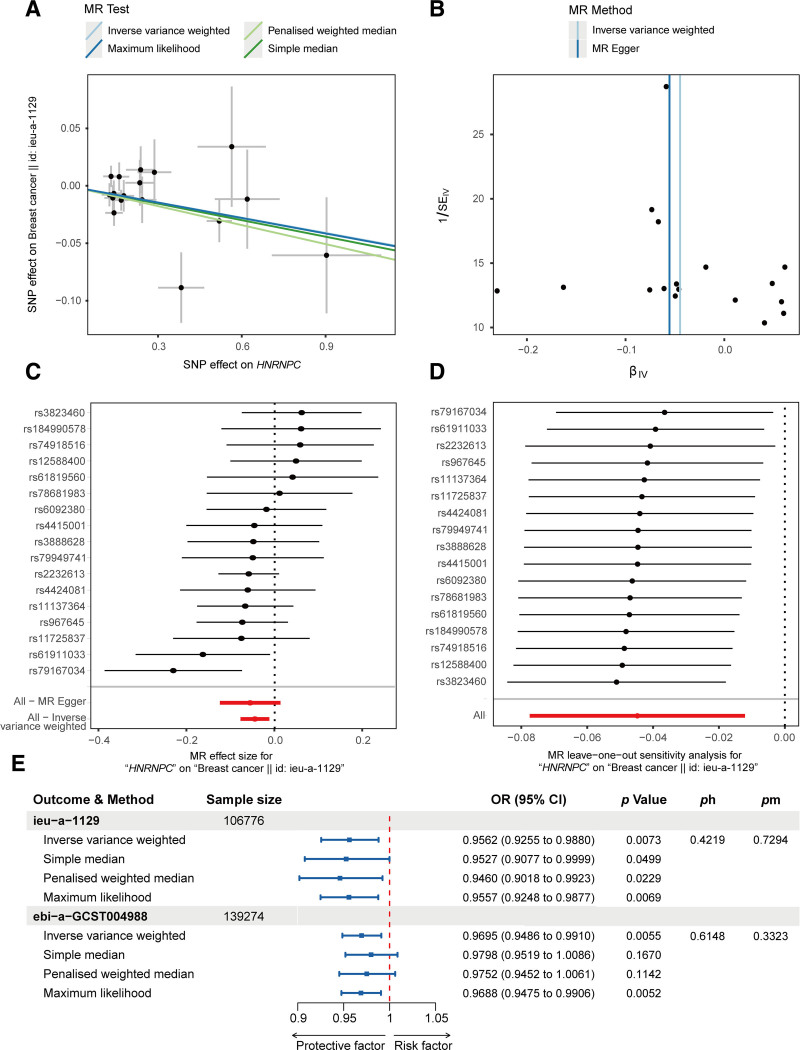
Result of two-sample MR analysis. (A) A scatter plot illustrating the effects of HNRNPC on breast cancer, with each point signifying an SNP among the 17 in the HNRNPC genetic tool, and the gradients reflecting the results of the 4 different MR analyses. (B) Funnel plots employed to evaluate MR heterogeneity for the impact of HNRNPC on breast cancer risk. The asymmetry of the plot is taken to be indicative of pleiotropy. (C) A forest plot displaying the causal influence of individual SNPs on breast cancer risk. (D) A leave-one-out plot illustrating the causal impact of HNRNPC on breast cancer risk, excluding a single SNP per iteration. (E) A forest plot summarizing the results of MR analysis for different outcome datasets and MR methods. Ph: the *P*-value for the heterogeneity test; Pm: the *P*-value for the MR-Egger intercept; Pp: the *P*-value for the MR-PRESSO global test. HNRNPC = heterogeneous nuclear ribonucleoprotein C, MR = Mendelian randomization, SNP = single-nucleotide polymorphism.

IDC has been established to be the predominant form of breast cancer,^[4,5]^ and several recent studies have focused on the complex interplay between *HNRNPC*, identified as a key gene in the risk signature described herein, and different aspects of breast cancer.^[56–59]^ On the basis of two-sample MR analysis, we identified 17 SNPs when using *HNRNPC* as the exposure and breast cancer as the outcome, and using the IVW method, we established a significant association between *HNRNPC* and breast cancer, with an odds ratio (OR) of 0.9562 [95% confidence interval (CI) = 0.9255–0.9880; *P* = .0073]. Moreover, the application of supplementary MR methods similarly revealed statistically significant effects as follows: simple median (OR = 0.9527, 95% CI = 0.9077–0.9999, *P* = .0499), penalized weighted median (OR = 0.9460, 95% CI = 0.9018–0.9923, *P* = .0229), and maximum likelihood (OR = 0.9557, 95% CI = 0.9248–0.9877, *P* = .0069) (Fig. 8). As depicted using a funnel plot, the causal effect was characterized by near perfect symmetry (Fig. 8B), and the intercept obtained for MR-Egger regression (0.0074 with a *P* value of .7294) indicated the absence of any evidence suggestive of horizontal pleiotropy, thereby reinforcing the credibility of the observed causal effects. Furthermore, heterogeneity tests revealed Q statistics of 16.3164 for MR-Egger with 15 degrees of freedom (*P* = .3613) and 16.4516 for the IVW method with 16 degrees of freedom (*P* = .4219), both similarly indicating the lack of any significant heterogeneity in the causal effects. In addition, leave-one-out analysis failed to reveal any significant issues, thus tending to indicate that no single SNP had a predominant influence on the association between *HNRNPC* levels and breast cancer (Fig. 8D). Repeating the analysis using a further GWAS dataset (Fig. 8E) similarly revealed that an elevated expression of *HNRNPC* is significantly associated with a heightened risk of IDC (OR = 0.9695, 95% CI = 0.9486–0.9880, *P* = .0055).

## 4. Discussion

IDC, the most commonly diagnosed subtype of breast cancer, is a complex multi-step process, for which, the findings of recent studies have highlighted the prominent role played by epigenetics, particularly m^6^A RNA modifications, in IDC development.^[32]^ Given the well-established heterogeneity of breast cancer, there is a clear need to delve deeper into the mechanisms whereby epigenetic differences influence the tumorigenesis and progression of the different subtypes.

Our findings in this study are broadly consistent with the existing body of research indicating that the development of IDC is associated with abnormal patterns of m^6^A RNA methylation.^[60]^ On the basis of an in-depth analysis of 656 patient samples compared with 81 normal samples derived from the TCGA database, we identified the significant up- or downregulation of 12 of the 17 assessed m^6^A RNA methylation regulators, thereby providing evidence of the potentially key roles played by these genes in the oncogenesis and progression of IDC. It is, however, important to emphasize that our findings differ from the those of previous studies on m^6^A RNA methylation in breast cancer, which have implicated *ALKBH5, FTO*, and *METTL3* as promoters of tumorigenesis and cancer cells.^[61–63]^ Our observations of notable disparities in the expression patterns of these 3 genes accordingly highlight the necessity for a more comprehensive investigation of such disparities in molecular expression among the different subtypes of a single cancer type. This also serves to emphasize the complexity of cancer biology and the importance of subtype-specific analyses to gain a more complete understanding of cancer progression and therapeutic responses. In this regard, with a view toward determining whether patients with IDC could be stratified into different subtypes based on the expression of m^6^A RNA methylation regulators, we performed consensus clustering analysis, on the basis of which, IDC samples were segregated into 2 risk-associated groups. However, with respect to overall survival, we detected no significant difference between patients in the 2 subsets, and the distribution of clinicopathological features was found to be independent of the subsets, which may be related to the consistent cluster grouping. Accordingly, this may indicate that m^6^A RNA methylation is not a suitable parameter for classification among the different types of cancer.

One of our primary objectives of the present study was to identify a method that could be used to precisely predict the prognosis of IDC. Using LASSO-Cox regression coupled with independent analyses, we established a 4-gene signature comprising *HNRNPC, YTHDF3, YTHDF2*, and *RBM15B*, which accurately predicts patient outcomes and was demonstrated to be an independent prognostic indicator based on analysis of a TCGA cohort of patients with IDC. Notably, for all assessed time points, this risk signature was established to have a superior discriminative performance when compared with previously developed IDC and breast cancer prognostic models.

When stratifying patients with IDC based on key clinical and pathological parameters, there is a tendency of an unfavorable prognosis for the high-risk group with respect to all assessed strata. However, the risk signature constructed based on the expression of m^6^A RNA methylation regulators was found to show a good correlation with overall survival, specifically regarding certain clinical and pathological characteristics, namely, AJCC stages 1 and 2, stages T1 and T2, stage M0, and a negative HER2 status. On the basis of these findings, we hypothesize that this risk signature may be more suitable for predicting the prognosis of patients at an early stage of IDC development than for those at later stages of the disease. Moreover, validation based on assessments using multiple independent GEO cohorts enabled us to confirm the robustness and generalizability of the risk signature among diverse populations, thus emphasizing its clinical reliability and broad applicability. However, further validation in more diverse populations would clearly contribute to establishing its generalizability.

The findings of recent studies have contributed to elucidating the multifaceted associations between multiple m^6^A RNA methylation regulators and IDC. To identify potential IDC biomarkers, we focused on the associations between the 4 genes constituting the risk signature and IDC, among which was HNRNPC, an essential RNA-binding protein that modulates mRNA expression via m^6^A recognition and has been established to play roles in cancer development and innate immunity.^[57,64]^ More specifically, it functions as a translational controller of a specific mRNA regulon, which is downregulated in cells that have metastasized, causing an extension of the 3′-untranslated regions (UTRs) of its bound mRNAs, thereby resulting in a suppression of their translation.^[65,66]^ Consequently, a reduction in the expression of *HNRNPC* and its regulon target tends to be correlated with adverse outcomes in patients with breast cancer, and, accordingly, manipulating the expression of *HNRNPC* could contribute to influencing the metastatic potential of breast cancer cells.^[67]^

Among the other signature proteins, excessive levels of YTHDF3 have been shown to be associated with the brain metastasis of breast cancer,^[68]^ enhancing cancer cell interactions with brain microvascular endothelial and astrocyte cells, as well as promoting blood–brain barrier permeability, angiogenesis, and invasion, thus resulting in reduced survival.^[69,70]^ The upregulation of *YTHDF2* in breast cancer cells stimulates the transcription of genes implicated in cell migration, wound healing, and metastasis.^[20]^ Mechanistically, YTHDF2 binds to m^6^A modifications in the 3′-UTR, resulting in a reduction in poly(A) tail length and mRNA decay, which may protect cells from endoplasmic reticulum stress and protein toxicity.^[71,72]^ *RBM15B* has been identified in prognostic models for a range of malignancies, including small cell lung cancer,^[73]^ esophageal cancer,^[74]^ and uveal melanoma^[75]^; however, its contribution to the progression of breast cancer is yet to be sufficiently established.

Drawing on previous research findings, by integrating expression profiles, survival analyses, and immunohistochemical staining images, we identified *HNRNPC* as the diagnostically most significant gene among the signature m^6^A RNA methylation regulators. To substantiate the link between the potential biomarker *HNRNPC* and IDC, we used GWAS data to conduct a two-sample MR analysis of *HNRNPC* and breast cancer, with a view toward confirming the causal relationship between *HNRNPC* expression and a heightened risk of IDC. The outcomes of the IVW method were established to be consistent with those of other supplementary MR techniques among multiple GWAS datasets, and sensitivity analyses failed to reveal any statistical evidence contravening the MR presuppositions.

Collectively, our findings in this study enabled us to establish a robust prognostic signature that is closely correlated with unfavorable clinical outcomes among patients with IDC, and we demonstrated a notable causal association between the expression of the key risk signature gene *HNRNPC* and a heightened risk of developing IDC. These findings accordingly provide evidence to indicate that the assessed m^6^A RNA methylation regulators may have a pronounced influence on the pathogenesis and progression of IDC, and thus could potentially serve as molecular biomarkers for monitoring IDC development, thereby contributing to the development of therapeutic strategies and drugs.

## 5. Conclusions

In this study, we successfully established a prognostic signature for IDC based on the identification of a 4-gene signature comprising *HNRNPC, YTHDF3, YTHDF2*, and *RBM15B*, which served as an independent prognostic factor for predicting patient outcomes. Our findings highlight the potential utility of m^6^A RNA methylation regulators as molecular biomarkers for the detection of IDC, with this signature being particularly informative for patients in which the disease is still at an early stage. The causal link established between *HNRNPC* expression and IDC risk based on MR analysis further highlights the significant roles played by these regulators in the pathogenesis of IDC. Building on the valuable insights gained in this study, future research should focus on external validation, functional studies, and assessing the applicability of the developed risk signature among different breast cancer subtypes and stages. The outcomes of such efforts will be essential for translating these findings into clinical practice, ultimately contributing to the development of more precise therapeutic strategies, and thus potentially enhancing the prognosis of patients with IDC.

## Acknowledgments

We are grateful for access to data from The Cancer Genome Atlas (TCGA), the Human Protein Atlas (HPA), and the Genome-Wide Association Studies (GWAS). The insights gained from these valuable resources were essential for the completion of this study. We would like to acknowledge the efforts of the TCGA Research Network, the HPA consortium, and the GWAS investigators for their contributions to the scientific community.

## Author contributions

**Conceptualization:** Dongshan Sun.

**Data curation:** Yibei Wang, Quhuan Li, Fengxia Zhang.

**Formal analysis:** Yibei Wang, Dongshan Sun, Ning Yang, Yue Shen.

**Funding acquisition:** Quhuan Li, Fengxia Zhang.

**Investigation:** Yibei Wang, Dongshan Sun, Ning Yang.

**Methodology:** Yibei Wang, Ying Kong.

**Project administration:** Yibei Wang, Quhuan Li.

**Resources:** Ying Kong, Fengxia Zhang.

**Software:** Ying Kong.

**Supervision:** Quhuan Li, Yue Shen.

**Validation:** Yibei Wang, Dongshan Sun, Yue Shen.

**Visualization:** Yibei Wang, Dongshan Sun.

**Writing – original draft:** Yibei Wang, Dongshan Sun.

**Writing – review & editing:** Quhuan Li, Fengxia Zhang.

## Supplementary Material


