# Supplementary material for: m6A RNA methylation regulator heterogeneous nuclear ribonucleoprotein C: A prognostic biomarker for invasive ductal carcinoma validated through Mendelian randomization and transcriptome analyses
Source: Medicine (Baltimore). 2025 Oct 10;104(41):e44733. doi: 10.1097/MD.0000000000044733 (PMC12517901; doi:10.1097/MD.0000000000044733)

Supplementary Figure 1. Comparison of the risk signature developed in this study with 19 previously developed prognostic models.

Receiver operating characteristic (ROC) curves of the risk signature (A), four invasive ductal carcinoma predictive models (B-E), ten breast cancer predictive models (F-O), and five triple-negative breast cancer predictive models (P-T).

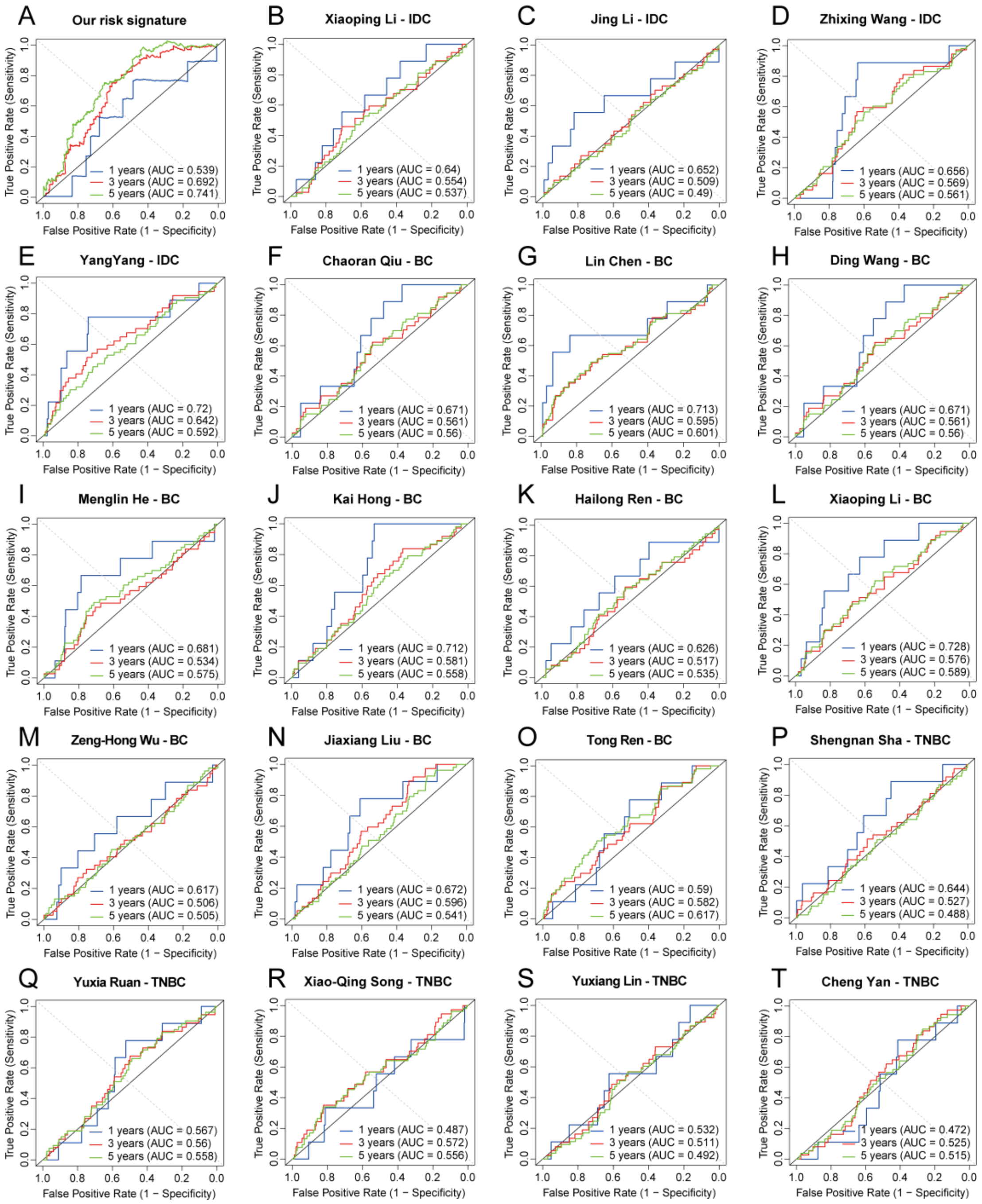

Supplementary Figure 2. External validation of the risk signature using three independent GEO cohorts.

(A-C) Kaplan–Meier survival curves showing significant stratification between high-risk (red) and low-risk (blue) groups in the GSE61304 (A), GSE42568 (B), and GSE7390 (C) cohorts. (D-F) Time-dependent receiver operating characteristic (ROC) curves showing predictive accuracy at 1-year (blue), 3-year (red), and 5-year (green) survival endpoints in the GSE61304 (D), GSE42568 (E), and GSE7390 (F) cohorts.

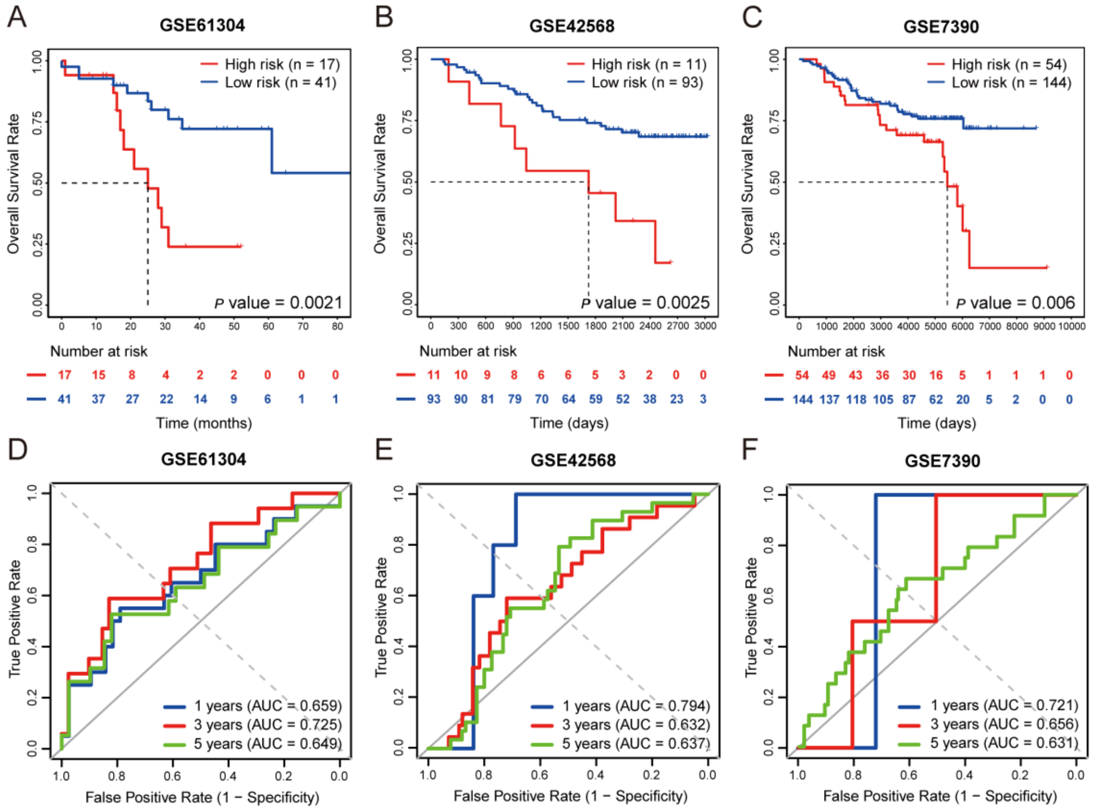

Supplement: Supplementary file 1 [file medi-104-e44733-s001.pdf]
